# Binocular visual performance and optical quality of trifocal intraocular lens in Chinese patients with high myopic cataract

**DOI:** 10.1371/journal.pone.0330473

**Published:** 2025-08-21

**Authors:** Baoxian Zhuo, Wenqian Shen, Lei Cai, Shuang Ni, Limei Zhang, Xu Chen, Haike Guo, Jiying Shen, Jin Yang

**Affiliations:** 1 Department of Ophthalmology and the Eye Institute, Eye and Ear, Nose, and Throat Hospital, Fudan University, Shanghai, China; 2 Key National Health Committee Laboratory of Myopia, Fudan University, and Laboratory of Myopia, Chinese Academy of Medical Sciences, Shanghai, China; 3 Shanghai Key Laboratory of Visual Impairment and Restoration, Shanghai, China; 4 Department of Ophthalmology, Shanghai Heping Eye Hospital, Shanghai, China; 5 Department of Ophthalmology, Shanghai Aier Eye Hospital, Shanghai, China; Saarland University, GERMANY

## Abstract

**Purpose:**

To evaluate the visual performance and optical quality of a trifocal intraocular lens (IOL) in patients with high myopic cataracts.

**Methods:**

This is a prospective, multicenter, non-randomized cohort study. Patients scheduled for cataract surgery with binocular AcrySof IQ® PanOptix® TFNT00 IOL implantation were enrolled. Participants with axial length (AL) ≥ 26 mm were assigned to the high myopia group, while age- and sex-matched patients with AL < 26 mm in the control group. Key outcomes measured included uncorrected distance visual acuity (UDVA), intermediate and near visual acuity (UIVA, UNVA), best-corrected distance visual acuity, defocus curve, contrast sensitivity, visual quality (objective and subjective), and reading ability.

**Results:**

58 eyes from 29 patients in the high myopia group and 56 eyes from 28 patients in the control group were included. Both groups showed significant improvements in visual acuity postoperatively although the UIVA and UNVA of patients with high myopia were significantly worse than those in the control group after 1 month, 3 months, and 1 year (both P < .05). The high myopia group had lower mean visual function-index scores for near-range activities and fewer photic phenomena (halo) than the control group (all P < .05). Preferred reading distance and speed did not differ significantly between the groups.

**C*onclusions*:**

Trifocal IOL implantation improved visual acuity in both groups. However, patients with high myopia experienced significantly poorer intermediate/near vision outcomes and also less complains about photic phenomena than controls. These findings support cautious patient selection and preoperative counseling for this population.

## Introduction

China bears a significant burden of myopia, with a prevalence of high myopia around 11.3% [1]. High myopia significantly increases the risk of cataracts, making patients more likely to develop cataracts and at an earlier age compared to the general population [2]. Meanwhile, high myopic cataract patients are characterized by the desire of spectacle-free and whole-course visual acuity. The rapid advancements in refractive cataract surgery have led to the development of multifocal intraocular lenses (IOLs), which offer promising solutions for these patients. However, it is still challenging to implant multifocal IOLs, such as trifocal IOLs, in high myopic cataract patients due to the higher frequency of intraoperative and postoperative complications arising from their fragile retinal conditions [3]. Careful preoperative examination and skilled surgical techniques are crucial. Nevertheless, even with these precautions and the exclusion of patients with retinopathies, high myopes typically experience suboptimal visual acuity and contrast sensitivity compared to low myopes [4,5]. High myopic patients, who are accustomed to near-range activities, have high demands for uncorrected near visual acuity (UNVA) and often report dissatisfaction with their postoperative UNVA. Due to a relative lack of research on trifocal IOL implantation, particularly the TFNT00 IOL, in patients with high myopia and cataracts, our study thus evaluated the visual outcomes and patient-reported optical quality of patients with high myopia and cataracts who underwent cataract surgery with TFNT00 IOL implantation.

## Methods

### Study design and participants

This multicenter, prospective, and cohort observational study was conducted at 3 sites in Shanghai, China: Eye and Ear, Nose, and Throat Hospital, Fudan University (from 12/01/2021 to 18/05/2022); Shanghai Heping Eye Hospital (from 13/04/2020 to 16/05/2022); and Shanghai Aier Eye Hospital (from 22/03/2021 to 27/06/2022). This research received approval from the Ethics Committees of the 3 hospitals and was conducted in adherence to the principles outlined in the Declaration of Helsinki (Trial Number: NCT05581888). All patients signed informed consent forms prior to participation.

The primary criteria for inclusion were patients with cataract, high demand for whole-course vision, and age ≥ 20 years. The main exclusion criteria were high myopic maculopathy (including atrophic, neovascular and tractional myopic maculopathy), preoperative corneal astigmatism of exceeding 1.0 D, excepted postoperative astigmatism less than 0.75D, previous ocular surgeries, progressively aggravated retinopathies (such as diabetic retinopathy and macular degeneration), anterior or posterior segment inflammation, expectation for secondary surgeries such as pars plana vitrectomy (PPV) with retinal reattachment surgery, IOL repositioning or exchange or neodymium-doped yttrium aluminum garnet (Nd:YAG) laser anterior capsulotomy within the follow-up period, and inability to maintain follow-up. Initially, we enrolled patients whose AL longer than 26 mm and were scheduled for cataract surgery and binocular AcrySof IQ^®^ PanOptix^®^ TFNT00 IOL implantation in the high myopia group. Age- and sex-matched non-myopic patients who has AL shorter than 26 mm were enrolled in the control group.

### Preoperative examination and surgical techniques

All the enrolled patients received a comprehensive ophthalmologic examination before surgery, including uncorrected distance visual acuity (UDVA) and best-corrected visual acuity (BCVA) measurements at 5 m using logMAR acuity charts under photopic conditions. Subjective refraction and slit-lamp examinations were conducted by an experienced ophthalmologist. Corneal topography was performed using Pentacam (Oculus Optikgeraete GmbH; Wetzlar, Germany). Ocular biometric parameters including anterior chamber depth, length thickness, and white-to-white distance were measured using the IOL-Master 700 (Carl Zeiss Meditec AG, Jena, Germany). The HOYA iTrace ray-tracing system (Tracey Technologies, Houston, TX, USA) was used to measure higher-order aberrations (HOAs), Strehl ratio (SR), and modulation transfer function (MTF) curve. Optical coherence tomography (Cirrus HD-OCT, Carl Zeiss Meditec, Dublin, CA), B-scan ultrasonography, optomap imaging (Optos Dayton, Carl Zeiss Meditec AG), and fundoscopy were also performed for every patient. All surgeries were performed by 3 experienced surgeons: (J.Y., HK.G., and X.C.) under topical anesthesia. The IOL power was calculated using the Barrett Universal II formula. ^6^ The targeted refractive outcome was emmetropia for the control group and a refractive error of −0.5 to 0 D for the high myopia group for BCVA [6,7]. A 2.2-mm transparent corneal incision was created, followed by phacoemulsification using the Centurion system (Alcon). Subsequently, the cortical material was irrigated and aspirated, and a trifocal IOL was then implanted into the capsular bag. For bilateral implantation, surgery was performed in the second eye 7 days after the first surgery. Postoperatively, anti-inflammatory and antibiotic agents were administered to the patients for 4 weeks.

### Outcome measurements

Clinical examinations were performed 1 day, 1 week, 1 month, 3 months, and 1 year after the implantation. UDVA, BCVA, uncorrected intermediate visual acuity (UIVA), UNVA, and subjective refraction were measured at each visit. Functional visual acuity reflected by contrast sensitivity (CS) and optical quality (defocus curves, HOAs, SR, and MTF curves) were assessed after 3 months of follow up. A questionnaire on the quality of vision (QoV) assessed the frequency and severity of optical disturbances. The modified Visual Function Index-14 (VF-14) recorded functional vision in relation to daily use, spectacle-independence (at distance, intermediate and near) and satisfaction about the whole-course vision. All examinations were conducted by 1 ophthalmic technician who was blinded to the study objective.

### Contrast sensitivity

Binocular contrast sensitivity was evaluated using an Optec6500 (Stereo Optical Co., Inc., Chicago, IL, USA) with best distance correction. The assessments were conducted across spatial frequencies of 1.5, 3, 6, 12, and 18 cycles per degree (cpd) with and without glare under both photopic (background luminance of 85 cd/m^2^) and mesopic conditions (background luminance of 3 cd/m^2^). A CS value refers to the value of the last patch that can be recognized. The CS score was transformed into logarithmic units, and log CS values were used for analyzing near reading ability.

### Reading ability

The Chinese reading visual acuity chart (CRVAC) was used to measure reading ability at near distance. Reading speed, expressed as words per minute (wpm) and preferred reading distance were measured using CRVAC. Only patients with UNVA > 0.1 logMAR underwent this examination. All available patients were instructed to read a text that included 30 test objects with a font size of 5 points (logMAR 0.5) at their preferred distance under the well-illuminated condition (400 ~ 480 Lux). The reading was timed while the reading distances were measured and recorded.

### Statistical analyses

Data analyses were performed using IBM SPSS Statistics software for Windows (version 26.0; IBM Corp., Armonk, NY, USA). All data from the right and left eye of each patient were highly-correlated (data not shown), therefore, the averaged values of both eyes were used to represent each patient. All continuous variables are expressed as mean ± standard deviation, and all categorical variables are expressed as numbers and percentages. Quantitative variables were assessed for normality using the Kolmogorov–Smirnov test. Students t-test or the Mann–Whitney U test was used for continuous variables, and the chi-square test was used for categorical variables. A *P* value of <.05 was considered statistically significant. Using PASS software for windows (version 15.0.5; NCSS Institute, Utah, USA), we calculated the sample size based on the available data to establish the statistically significance between 2 groups. The analysis indicated a minimum requirement of 12 patients per group, resulting in a total of 24 patients being included in the study (alpha = 0.05, power = 0.95).

## Results

### Demographic and preoperative characteristics

**Fig 1:**
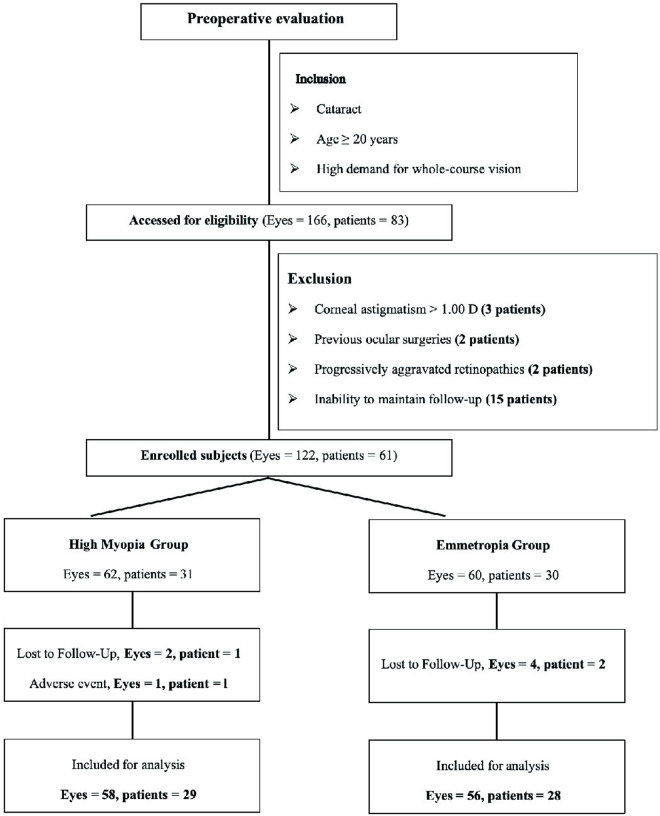
Enrollment and follow-up flow chart.

Initially, 122 eyes from 61 patients were included in the 2 groups. However, 2 patients in the control and 1 patient in high myopia group were lost to follow-up. Intraoperative complication was observed in 1 eye in the high myopia group (Fig 1). Finally, 58 eyes of 29 patients in the high myopia and 56 eyes of 28 patients in the control groups were included. Demographic and preoperative characteristics of the patients in the 2 groups were presented in Table 1. There were no significant differences in age or sex between the 2 groups. The ALs in the high myopia group were significantly longer than those in the control group (27.69 ± 1.05 mm vs. 23.75 ± 1.13 mm, *P* < .001). The high myopia group had a significantly deeper anterior chamber depth and larger white-to-white than those in the control group. Moreover, preoperative logMAR BCVA in the high myopia group was significantly worse than that in the control group (0.29 ± 0.18 vs. 0.18 ± 0.12, *P* = .038). Other characteristics such as preoperative intraocular pressure and the magnitude of corneal astigmatism were not significantly different between the 2 groups. The predicted SE was significantly different between the 2 groups (−0.09 ± 0.11 D and −0.23 ± 0.19 D for the control and high myopia groups, respectively (*P* = .015) since we reserved slight negative diopter for high myopia patients owing to the possibility of postoperative hyperopic shift in this population.

**Table 1:** Baseline parameters of the participants in the control and high myopia groups.

Variables	Overall	Control group (N = 28)	High myopia group (N = 29)	P value
Age (years)				0.169
Mean ± SD	57.64 ± 9.08	59.25 ± 8.50	56.10 ± 9.50	
Range	40-75	40-73	40-75	
Sex, number (%)				0.896
Male	28(49.1%)	14(50.0)	14(48.3)	
Female	29(50.9%)	14(50.0)	15(51.7)	
Axial length (mm)	25.75 ± 2.25	23.75 ± 1.13	27.69 ± 1.05	<0.001*
Intraocular pressure	14.34 ± 2.22	14.01 ± 1.93	14.54 ± 2.55	0.601
Corneal astigmatism (D)	0.63 ± 0.24	0.58 ± 0.24	0.68 ± 0.24	0.046*
Pre BCVA (LogMAR)	0.24 ± 0.16	0.18 ± 0.12	0.29 ± 0.18	0.038*
ACD (mm)	3.29 ± 0.37	3.12 ± 0.39	3.46 ± 0.28	<0.001*
LT (mm)	4.40 ± 0.36	4.43 ± 0.42	4.37 ± 0.29	0.202
WTW (mm)	11.91 ± 0.43	11.76 ± 0.45	12.05 ± 0.35	0.014*
Predicted spherical equivalent (D)	–0.15 ± 0.16	–0.09 ± 0.11	–0.23 ± 0.19	0.015*

D, diopter; Pre BCVA, preoperative best-corrected distance visual acuity; LogMAR, logarithm of the minimum angle of resolution; ACD, anterior chamber depth; LT, length thickness; WTW, white-to-white. Continuous variables were expressed as Mean ± SD. *P < 0.05.

### Visual acuity and defocus curve

**Fig 2:**
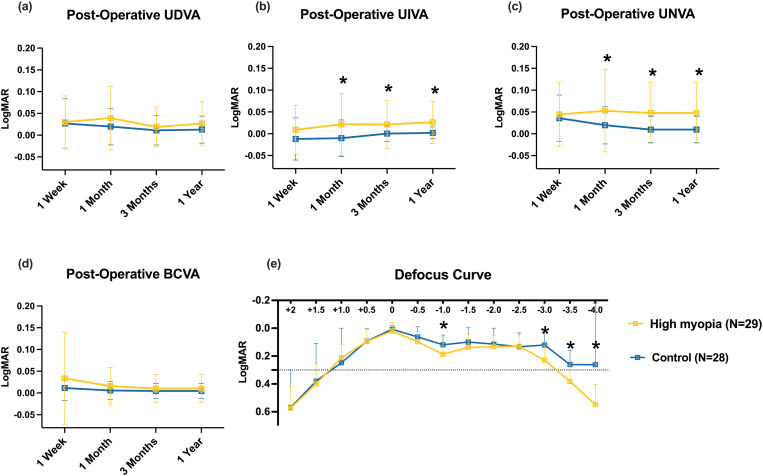
Postoperative outcomes of visual acuity and binocular defocus curves at 1 week, 1 month, 3 months, and 1 year. *P < 0.05.

The UDVA and BCVA did not differ significantly between the 2 groups at each postoperative visit (Fig 2). Moreover, the differences in UIVA and UNVA between control and high myopia groups were significant after 1 month, 3 months, and 1 year (−0.01 ± 0.04 vs. 0.02 ± 0.07, 0 ± 0.02 vs. 0.02 ± 0.05, 0.00 ± 0.01 vs. 0.03 ± 0.05, respectively for UIVA and 0.02 ± 0.04 vs. 0.05 ± 0.09, 0.01 ± 0.03 vs. 0.05 ± 0.07, 0.01 ± 0.03 vs. 0.05 ± 0.07, respectively for UNVA, P ≤ .05). According to the defocus curves, 2 peaks of maximal visual acuity were observed in both groups. After 3 months, patients with high myopia had significantly worse defocus levels of −1.0 D, −3.0 D, −3.5 D, and −4.0 D (P = .02, P = .002, P = .006 and P = .039 respectively). Moreover, the defocus curve showed that TFNT00 IOL can provide a visual acuity of ≥ 0.3 logMAR between defocus levels of +1.00 and −3.0 D in the high myopia group. The detailed data was provided in supplementary material 1.

## Objective visual quality and complications

HOAs were observed after 3 months of follow-up using an iTrace aberrometer (Supplementary material 2). No significant differences were found between the 2 groups in terms of HOAs, total coma, spherical aberrations, or trefoil. The corneal, internal, and total SR of the 2 groups were not significantly different. Similar negative results were observed for the MTF at 10 and 30 cpd.

### Contrast sensitivity

**Fig 3:**
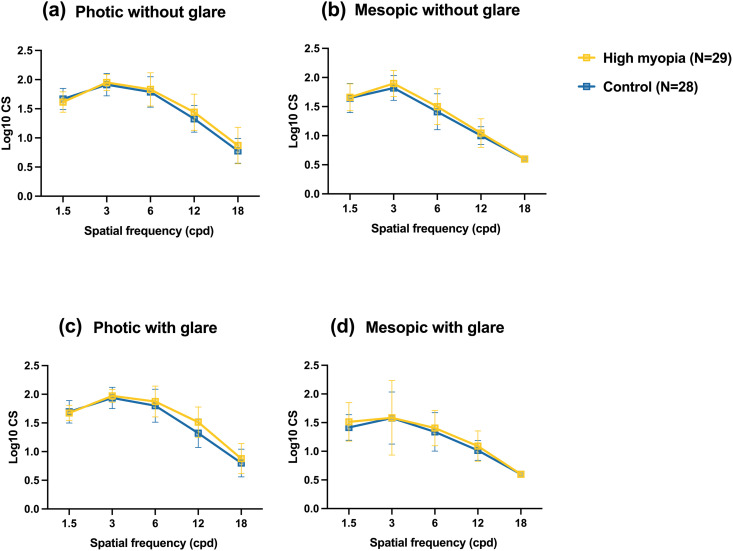
Binocular contrast sensitivity curves after trifocal intraocular lens implantation. Binocular contrast sensitivity data at different spatial frequencies were obtained without glare under photopic (a) and mesopic (b) conditions or with glare under photopic (c) and mesopic (d) conditions.

The results for binocular contrast sensitivity (CS) in both groups are presented in Fig 3. Peak CS performance was at a spatial frequency of 3 cpd under both photopic and mesopic conditions, with or without glare, and declined as spatial frequency increased (Fig 3 a–d). There were no significant differences in CS between the groups at any spatial frequency (*P* > .05).

### Subjective visual quality

#### Photic phenomena.

**Fig 4:**
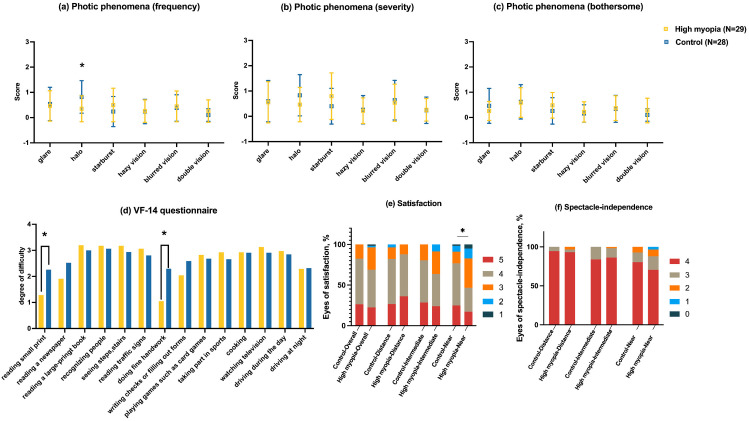
Results of the subjective visual quality. Level of photic phenomena between the 2 groups based on frequency (a), severity (b), and bothersome (c). Results of the VF-14 questionnaire for the control and high myopia groups (d). Satisfaction (e) and spectacle-independence (f) or whole-course vision. Satisfaction was assessed on a scale of 1 to 5, with 1 representing “extremely unsatisfied,” 2 “unsatisfied,” 3 “acceptable,” 4 “satisfied,” and 5 “completely satisfied.” Spectacle-independence was similarly evaluated on a scale of 0 to 4, where 0 indicated “always needing glasses,” 1 “often,” 2 “usually,” 3 “sometimes,” and 4 “never needing glasses.” *P < 0.05. VF-14, visual function index-14.

Clinically, glare, halo, starburst, haziness, blurring, and double vision are often complaints of photic phenomena. Frequency, severity, and bothersome characteristics were investigated using a questionnaire after 3 months of follow-up (Fig 4a, b, c). Overall, only the frequency of halos between the 2 groups was significantly different, showing a lower chance of patients in the high myopia group suffering from halos (*P* = .005).

#### VF-14 questionnaire.

The VF-14 questionnaire-based investigation was conducted 3 months after surgery (Fig 4d). The final score (25 × average score of 14 items) was not significantly different between high myopia and control groups (64.26 ± 13.97 vs. 67.53 ± 15.64; *P* = .733). The comparison of each item of the VF-14 showed significant differences in reading small prints and doing fine handwork (*P* = .002 and *P* < .001).

#### S*atisfaction and spectacle-independence for whole-course vision.*

Comparative analysis revealed high satisfaction rates across visual distance, with no statistically significant differences observed in most categories. Overall and distance vision satisfaction demonstrated comparable rates between high myopia and control group (96.55% vs. 100%, *P* = .317), as did intermediate vision (94.64% vs. 100%, *P* = .091), while near vision showed a statistically significant differential (93.10% vs. 98.28%, *P* = .015) (Fig 4e). Spectacle independence analysis similarly indicated equivalent performance in high myopia and control group for distance vision (96.55% vs. 100%, *P* = .832) and intermediate vision (98.28% vs. 100%, *P* = .552), with near vision independence rates approaching statistical parity (87.93% vs. 92.86%, *P* = .374) (Fig 4f).

### Reading acuity

Table 2 lists the near reading ability of patients in high myopia group and control group measured using CRVAC under photopic conditions. There was no significance in reading speed (216.86 ± 87.88 wpm vs. 244.82 ± 101.07 wpm) and the preferred distances for near vision (34.3 ± 3.70 cm vs. 35.7 ± 3.46 cm) between the control and high myopia groups.

**Table 2:** Reading acuity at near under photopic conditions.

Parameter, Mean ± SD	Control group (N = 28)	High myopia group (N = 29)	P value
Words per minute at preferred distance			
Near, 0.5 logMAR	216.86 ± 87.88	244.82 ± 101.07	0.689
Preferred reading distance			
Near	34.3 ± 3.70	35.7 ± 3.46	0.347

LogMAR, logarithm of the minimum angle of resolution.

There were no serious complications occurred except for posterior capsule opacity (PCO) during the 1-year follow-up. Only 1 patient (3.4%) in the high myopia group required YAG laser posterior capsulotomy to restore the original visual acuity.

## Discussion

The increasing prevalence of high myopia and its association with cataract development have spurred rapid advancements in refractive cataract surgery. For patients with high myopia, trifocal IOLs offer a feasible solution [8]. However, surgeons have reservations about using trifocal IOLs in patients with high myopia with cataracts due to uncertain effects [3,9]. Therefore, this prospective study investigated the efficacy of trifocal IOL implantation in patients with high myopia and compared postoperative outcomes between patients with and without high myopia.

Overall, we found that after trifocal IOL implantation, the UIVA and UNVA of patients with high myopia is significantly worse than those without. We revealed superior UNVA in the control cohort (mean 0.02 logMAR) compared to established benchmarks (0.08–0.14 logMAR), while the high myopia group demonstrated comparable UNVA performance (0.05 logMAR) to previous trials utilizing identical intraocular lens (IOL) implantation protocols [10,11]. Regarding uncorrected distance visual acuity (UDVA), both cohorts exhibited remarkable consistency with historical data: the control group achieved 0.01 logMAR (current findings) versus 0.003–0.014 logMAR in prior investigations, and the high myopia group matched published IOL-specific results at 0.02 logMAR [10,12]. Since the implementation of rigorous preoperative fundus screening protocols coupled with systematic refractive error optimization, both two groups achieved clinically significant improvements in postoperative visual acuity at far, intermediate, and near distances. However, between-group comparison of VA showed a similar UDVA but a significant better UIVA and UNVA in the control group than in the high myopia group at each visit expect 1-week. In a study involving 10 patients undergoing trifocal intraocular lens (IOL) implantation within a 0.0–10.0 diopter range, Steinwender et al. documented postoperative visual outcomes of 0.12 ± 0.04, 0.13 ± 0.05, and 0.06 ± 0.03 logMAR for UNVA, UIVA, and UDVA, respectively [13]. Although our visual outcomes displayed more favorable trends compared to Steinwender’s report, it was potentially attributable to the limited representation of extreme long eyes in our cohort. Notably, the high myopia cohort exhibited statistically significant inferiority in intermediate and near visual performance versus controls, with this comparative analysis objectively revealing compromised trifocal intraocular lens (IOL) efficacy in high myopia patients relative to emmetropia ones. The difference was further confirmed by the postoperative defocus curves of the two groups.

The defocus curves of the control group and the high myopia group in our study were respectively similar to those of emmetropic patients and high myopia patients in previous studies [14]. Both groups showed a good continuity in VA between +0.5 D and −2.5 D, illustrating the TFNT00 IOL can provide outstanding continuous VA from far to near distance in daily activities. However, comparative defocus curve analysis demonstrated significantly superior VA in the control group compared to the high myopia group at −1.0 D and throughout the −3.0 to −4.0 D defocus range, with this functional disparity clinically correlating to postoperative adaptive behaviors in high myopia patients including increased reading distances, reduced intermediate/near vision satisfaction scores, and increased dependence on spectacles. This aligns with Liu’s observation of better near and intermediate visual acuity on defocus curve (from −1.5 D to −3.0 D) of the AL < 26 mm compared with AL > 28 mm group after another trifocal diffractive IOLs (AT LISA tri 839MP) implantation [8].

This phenomenon may stem from structural alterations in high myopia with AL ≥ 26.0 mm, characterized by abnormal posterior pole retinal pigment epithelium (RPE) cell density gradients (foveal ≤12,500 cells/mm² vs normal ≥15,000 cells/mm² via widefield fundus autofluorescence imaging), reduced choriocapillaris flow density, and shortened photoreceptor outer segment (POS) length [15–17]. These changes collectively impair high spatial frequency resolution through decreased light capture efficiency and metabolic support to photoreceptors. According to Knapp’s law, each −1.00D myopia induces 1.5% retinal image magnification, which compensates for POS shortening-induced acuity reduction by enhancing spatial sampling density of the magnified retinal image [18]. This magnification effect enables superior near-task performance in uncorrected high myopes [19]. However, IOL implantation eliminates this compensatory magnification while simultaneously increasing near-focusing distance. Although improving distance VA, this dual effect reduces retinal image size below the critical sampling threshold for small text recognition, explaining the paradoxical results in decreased score of high myopia group in reading small prints and doing fine handwork in VF-14 despite equivalent reading ability [20].

Critically, our data indicate that patients with high myopia experience significantly worse intermediate and near visual performance with trifocal IOLs compared to non-highly myopic controls. This underscores the necessity for comprehensive preoperative counseling in this population, specifically addressing potential limitations in functional vision at intermediate/near ranges. Surgeons should weigh these outcomes against potential benefits when considering trifocal IOLs for high myopia patients.

Although it had no significant differences of the between-group comparison in reading ability including reading speed and preferred reading distance, it did not mean the two group of patients had the same reading performance. In one hand, our assessments were conducted by the patients with UIVA/UNVA >0.1 logMAR under well-illuminated conditions; and in the other hand, given the high myopia patients’ threshold print size of 5-point font, the control cohort was subjected to equivalent 5-point font assessments to ensure comparative validity. While normative visual systems typically demonstrate smaller minimum resolvable print sizes, this controlled protocol intentionally eliminated potential confounding variables, though subsequent analysis of subthreshold fonts (≤3-point) would likely demonstrate significant intergroup disparities in reading ability.

Furthermore, the prevalence of diffractive ring designs in trifocal IOLs has sparked growing debate among surgeons about their association with clinically significant photic disturbances under mesopic conditions after implantation. Interestingly, the results of the questionnaire on photic phenomena revealed that a higher percentage of patients in the control group reported experiencing halos than those in the high myopia group (75.0% vs. 44.4%, P = .005). Nevertheless, the relatively minor inconvenience reported by patients in both groups regarding photic phenomena suggests that these disturbances did not significantly disrupt their daily lives. The main reason of this result was that high myopia eyes usually had reduced photoreceptor density in the RPE and enhanced Müller cell-mediated scatter light filtration, leading to diminished subjective perception of halos and glare in this population [8]. Previous study of wavefront aberrometry analyses corroborated this phenomenon, showing significantly higher total HOA root mean square (RMS) values in high myopia eyes than in shorter axial lengths postoperatively [21]. However, the MTF curve maintained steeper slopes within 5–15 cycles/degree ranges, indicating superior tolerance to elevated spatial frequency distortions in high myopia [22]. These findings suggest that structural and functional adaptations in the myopic retina mitigated the perceptual impact of photic phenomena.

This study has several limitations that should be addressed in future studies. First, as a subjective outcome, individual characteristics may have influenced the reliability of the questionnaire. Second, the follow-up duration was short. Further multicenter investigations with prolonged follow-up periods are necessary to investigate the generalizability and broad application of our findings concerning binocular trifocal IOLs implantation in individuals with high myopia.

In conclusion, although trifocal IOL implantation in patients with high myopic cataract can achieve overall satisfactory visual results with fewer photic phenomena, there are deficiencies in the performance of near-range activities and fine object recognition in these patients. Under good patient expectation management, the trifocal artificial IOL are able to meet the daily visual demands of high myopic patients with cataracts and normal fundus conditions.

## Supporting information

Supplementary Material 1 Postoperative visual acuity and refractive outcomes. (DOCX)

Supplementary Material 2 Comparison of postoperative high-order aberrations (HOAs), strehl ratio (SR), and modulation transfer function (MTF) 3 Months after surgery in 114 eyes of 57 patients. (DOCX)
