# Supplementary material for: Binocular visual performance and optical quality of trifocal intraocular lens in Chinese patients with high myopic cataract
Source: PLoS One. 2025 Aug 21;20(8):e0330473. doi: 10.1371/journal.pone.0330473 (PMC12370134; doi:10.1371/journal.pone.0330473)
Supplement: Supplementary Material 1 — (DOCX) [file pone.0330473.s001.docx]

| Variables | Overall | Control group  (N=28) | High myopia group (N=29) | P value |
| --- | --- | --- | --- | --- |
| Post 1 week |  |  |  |  |
| UDVA | 0.02 ± 0.05 | 0.03 ± 0.06 | 0.03 ± 0.06 | 0.775 |
| UIVA | -0.0 ± 0.05 | -0.01 ± 0.05 | 0.01 ± 0.06 | 0.051 |
| UNVA | 0.04 ± 0.06 | 0.04 ± 0.05 | 0.04 ± 0.07 | 0.499 |
| BCVA | 0.02 ± 0.10 | 0.01 ± 0.03 | 0.03 ± 0.15 | 0.29 |
| Post 1 month |  |  |  |  |
| UDVA | 0.02±0.05 | 0.02 ± 0.04 | 0.04 ± 0.07 | 0.111 |
| UIVA | 0.00 ± 0.05 | -0.01 ± 0.04 | 0.02 ± 0.07 | 0.009* |
| UNVA | 0.03 ± 0.07 | 0.02 ± 0.04 | 0.05 ± 0.09 | 0.031* |
| BCVA | 0.01 ± 0.03 | 0.01 ± 0.02 | 0.02 ± 0.04 | 0.151 |
| Post 3 months |  |  |  |  |
| UDVA | 0.01 ± 0.03 | 0.01 ± 0.03 | 0.02 ± 0.05 | 0.342 |
| UIVA | 0.01 ± 0.04 | 0 ± 0.02 | 0.02 ± 0.05 | 0.014* |
| UNVA | 0.02 ± 0.05 | 0.01 ± 0.03 | 0.05 ± 0.07 | 0.001* |
| BCVA | 0.00 ± 0.02 | 0 ± 0.02 | 0.01 ± 0.03 | 0.296 |
| SE | -0.18 ± 0.34 | -0.12 ± 0.29 | -0.22 ± 0.29 | 0.390 |
| Post 1 year |  |  |  |  |
| UDVA | 0.01 ± 0.04 | 0.01 ± 0.03 | 0.03 ± 0.05 | 0.099 |
| UIVA | 0.01 ± 0.03 | 0 ± 0.01 | 0.03 ± 0.05 | 0.001* |
| UNVA | 0.02 ± 0.05 | 0.01 ± 0.03 | 0.05 ± 0.07 | 0.001* |
| BCVA | 0.00 ± 0.02 | 0 ± 0.02 | 0.01 ± 0.03 | 0.296 |
| UDVA: Uncorrected Distance Visual Acuity; UIVA: Uncorrected Intermediate Visual Acuity; UNVA: Uncorrected Near Visual Acuity; BCVA: Best Corrected Visual Acuity; SE: Spherical Equivalent | | | | |
