# Supplementary material for: Binocular visual performance and optical quality of trifocal intraocular lens in Chinese patients with high myopic cataract
Source: PLoS One. 2025 Aug 21;20(8):e0330473. doi: 10.1371/journal.pone.0330473 (PMC12370134; doi:10.1371/journal.pone.0330473)
Supplement: Supplementary Material 2 — (DOCX) [file pone.0330473.s002.docx]

Supplementary material 1. Comparison of postoperative high-order aberrations (HOAs), strehl ratio (SR), and modulation transfer function (MTF) 3 Months after surgery in 114 eyes of 57 patients

| Variables | Overall | Control group (N=28) | High myopia group (N=29) | *P* value |
| --- | --- | --- | --- | --- |
| HOA total | 0.14 ± 0.06 | 0.16 ± 0.08 | 0.13 ± 0.05 | 0.629 |
| HOA total coma (μ) | 0.06 ± 0.03 | 0.06 ± 0.04 | 0.06 ± 0.03 | 0.469 |
| HOA total sph (μ) | 0.00 ± 0.02 | 0.01 ± 0.02 | 0.00 ± 0.01 | 0.493 |
| HOA total sa (μ) | 0.03 ± 0.02 | 0.03 ± 0.02 | 0.03 ± 0.02 | 0.392 |
| HOA total trefoil (μ) | 0.08 ± 0.04 | 0.09 ± 0.03 | 0.07 ± 0.04 | 0.13 |
| HOA inter | 0.12 ± 0.06 | 0.14 ± 0.08 | 0.11 ± 0.05 | 0.345 |
| HOA cornea | 0.07 ± 0.04 | 0.07 ± 0.03 | 0.07 ± 0.04 | 0.903 |
| SR total | 0.15 ± 0.06 | 0.13 ± 0.06 | 0.16 ± 0.06 | 0.237 |
| SR inter | 0.22 ± 0.09 | 0.21 ± 0.12 | 0.23 ± 0.08 | 0.48 |
| SR cornea | 0.25 ± 0.13 | 0.25 ± 0.09 | 0.25 ± 0.15 | 0.406 |
| MTF-10 total | 0.39 ± 0.10 | 0.37 ± 0.14 | 0.40 ± 0.08 | 0.331 |
| MTF-10 inter | 0.47 ± 0.12 | 0.43 ± 0.16 | 0.49 ± 0.08 | 0.3 |
| MTF-10 cornea | 0.56 ± 0.12 | 0.58 ± 0.12 | 0.55 ± 0.13 | 0.318 |
| MTF-30 total | 0.10 ± 0.03 | 0.09 ± 0.03 | 0.11 ± 0.04 | 0.177 |
| MTF-30 Inter | 2.14 ± 11.1 | 0.14 ± 0.09 | 0.15 ± 0.07 | 0.354 |
| MTF-30 Cornea | 0.17 ± 0.08 | 0.19 ± 0.08 | 0.16 ± 0.08 | 0.261 |
| HOAs: high-order aberrations, SR: strehl ratio, MTF: modulation transfer function | | | | |
